# Accuracy of Death Certificates and Assessment of Factors for Misclassification of Underlying Cause of Death

**DOI:** 10.2188/jea.JE20150010

**Published:** 2016-04-05

**Authors:** Makiko Naka Mieno, Noriko Tanaka, Tomio Arai, Takuya Kawahara, Aya Kuchiba, Shizukiyo Ishikawa, Motoji Sawabe

**Affiliations:** 1 Department of Medical Informatics, Center for Information, Jichi Medical University, Shimotsuke, Tochigi, Japan; 1 自治医科大学 情報センター・医学情報学; 2 Biostatistics Section, Department of Clinical Research and Informatics, Clinical Research Center, National Center for Global Health and Medicine, Tokyo, Japan; 2 国立国際医療研究センター臨床研究センター医療情報解析研究部医学統計研究室; 3 Department of Pathology, Tokyo Metropolitan Geriatric Hospital, Tokyo, Japan; 3 東京都健康長寿医療センター病理診断科; 4 Department of Biostatistics, School of Public Health, Graduate School of Medicine, the University of Tokyo, Tokyo, Japan; 4 東京大学大学院医学系研究科公共健康科学専攻生物統計学教室; 5 Department of Biostatistics, National Cancer Center, Tokyo, Japan; 5 国立がん研究センター研究支援センター生物統計部; 6 Center for Community Medicine, Jichi Medical University, Shimotsuke, Tochigi, Japan; 6 自治医科大学 医学教育センター; 7 Section of Molecular Pathology, Graduate School of Health Care Sciences, Tokyo Medical and Dental University, Tokyo, Japan; 7 東京医科歯科大学保健衛生学研究科生体検査科学専攻分子病態検査学講座

**Keywords:** accuracy, autopsy, death certificates, outcome misclassification, underlying cause of death

## Abstract

**Background:**

Cause of death (COD) information taken from death certificates is often inaccurate and incomplete. However, the accuracy of Underlying CODs (UCODs) recorded on death certificates has not been comprehensively described when multiple diseases are present.

**Methods:**

A total of 450 consecutive autopsies performed at a geriatric hospital in Japan between February 2000 and August 2002 were studied. We evaluated the concordance rate, sensitivity, and specificity of major UCODs (cancer, heart disease, and pneumonia) reported on death certificates compared with a reference standard of pathologist assessment based on autopsy data and clinical records. Logistic regression analysis was performed to assess the effect of sex, age, comorbidity, and UCODs on misclassification.

**Results:**

The concordance rate was relatively high for cancer (81%) but low for heart disease (55%) and pneumonia (9%). The overall concordance rate was 48%. Sex and comorbidity did not affect UCOD misclassification rates, which tended to increase with patient age, although the association with age was also not significant. The strongest factor for misclassification was UCODs (*P* < 0.0001). Sensitivity and specificity for cancer were very high (80% and 96%, respectively), but sensitivity for heart disease and pneumonia was 60% and 46%, respectively. Specificity for each UCOD was more than 85%.

**Conclusions:**

Researchers should be aware of the accuracy of COD data from death certificates used as research resources, especially for cases of elderly patients with pneumonia.

## Abstract (ja)

**背景：:**

死亡票から得られた死因はしばしば不正確かつ不完全である。しかしながら、死亡票に記録された原死因の正確性について、特に多疾患を含む場合には網羅的に検討されてきていない。

**方法：:**

日本国内のある老年病専門病院で2000年2月から2002年8月に行われた連続剖検450例を対象とした。死亡票で報告された主な原死因（悪性腫瘍、心疾患、肺炎）を、剖検データと臨床カルテ記録に基づいて病理学者が判断した参照基準と比較し、一致率、感度、特異度について評価した。性、年齢、併存疾患、原死因の誤分類への影響を評価するため、ロジスティック回帰分析を行った。

**結果：:**

悪性腫瘍についての一致率は81%と比較的高い結果であったが、心疾患では55%、肺炎では9%と低い一致率を示した。全体の一致率は48%であった。性と併存疾患は原死因の誤分類率に影響しなかった。また、統計的有意ではなかったものの、患者年齢が上がるにつれて誤分類率は上昇する傾向がみられた。誤分類に対するもっとも強い要因は原死因であった(p<0.0001)。悪性腫瘍についての感度は80%、特異度は96%と高かった一方で、心疾患と肺炎の感度はそれぞれ60%と46%であった。各原死因についての特異度は85%以上を示していた。

**結論：:**

研究者は、高齢の肺炎患者を対象とした場合は特に、研究資源として用いた死亡票の死因データの正確性に注意を払うべきである。

## INTRODUCTION

Cause of death (COD) data from death certificates are often used in epidemiological studies to estimate mortality rates or risk of death from certain diseases. However, the accuracy and utility of COD data from death certificates are uncertain and often questionable.^1^^–^^5^ For cancer mortality statistics in particular, uncertainty regarding the information on death certificates has been discussed for more than 100 years. For example, in early 1900s, Riechelmann reported differences in the number of cancer cases between autopsy and vital statistics reports,^6^ and Wells discussed the degree of this influence on vital statistics.^7^ In the late 20th century, Hoel et al reviewed the effect of death certificate error on cancer mortality statistics and found a consistent 18% underestimation of total cancer mortality, with an especially large influence on the elderly population (75 years or older).^8^ Since around 2000, site-specific analyses for misclassification have been investigated. For example, Percy et al reported on misclassification in colorectal cancer, finding that colon cancer was over-reported while rectal cancer was underreported on death certificates.^9^ Similarly, Yin et al indicated that 82% of misclassified rectal cancer deaths were coded as colon cancer deaths.^10^

For diseases other than cancer, Cheng et al reported death certificate sensitivity and specificity for diabetes of 34.7% and 98.1%, respectively. In their 30-year study, they also reported cardiovascular disease-related diabetes sensitivity stratified by decade of death and showed a time trend of improved sensitivity that reflected increased recognition of cardiovascular disease risk factors.^11^ In Japan, Saito et al reported the validity of death certificates for ischemic heart diseases after the ICD-10 code revision. They compared death certificates and the diagnosis examined by a review of the medical records and/or interviews with physicians and reported that the sensitivity and specificity for ischemic heart disease certified as the cause of death was 86.5% and 64.7%, respectively.^12^ Ravakhah compared death certificate diagnoses with autopsy report diagnoses in 223 cases and reported that myocardial infarction was more likely to be unsuspected in women and those with advanced age.^13^ Kohn reviewed autopsy findings in 200 persons older than 85 years, indicating that the autopsy data were in strong disagreement with the causes of death listed in the vital statistics and proposing that ‘senescence’ be accepted as a cause of death.^14^

These studies underscore the difficulty in specifying underlying COD (UCOD), especially among elderly people, who tend to have multiple diseases before death. However, the accuracy of UCODs recorded on the death certificates of elderly people has not yet been comprehensively examined for multiple diseases using consecutive autopsy studies. Here, we evaluated the accuracy of UCODs of elderly people recorded on death certificates compared to a reference standard of autopsy findings.

## METHODS

### Study subjects

Of 532 consecutive autopsies performed at the Tokyo Metropolitan Geriatric Hospital (Tokyo, Japan) between February 2000 and August 2002, 450 (84.6%) were included in the present study. No medico-legal cases were included. The average autopsy rate during this period was 32%. All subjects were registered in the geriatric autopsy database (GEAD) at the Tokyo Metropolitan Geriatric Hospital, which contains clinical information (presence or absence of 26 geriatric diseases, as follows: ischemic heart disease, atrial fibrillation, degenerative valvular diseases, hypertension, aneurysm, arteriosclerosis obliterans, dementia, cerebrovascular disorder, Parkinson’s disease, diabetes mellitus, hyperlipidemia, malnutrition, osteoporosis, degenerative osteoarthritis, aspiration, chronic obstructive pulmonary disease, idiopathic interstitial pneumonia, urinary tract infection, prostatic hypertrophy, decubital ulcer, lung cancer, gastric cancer, colon cancer, hematopoietic malignancy, cataract, and glaucoma, as well as clinical dementia ratings and histories of smoking and alcohol consumption) and pathological findings (720 items frequently encountered in autopsy examinations of elderly subjects). Details on the GEAD have been reported elsewhere.^15^

### COD data

All CODs recorded on death certificates based on clinical and autopsy records were first evaluated by M.S., a pathologist and co-author of this study, for reporting consistency and adherence to instructions for proper completion of the death certificate. The CODs were subsequently evaluated by T.A., also a pathologist and co-author of this study, to confirm the accuracy of the findings and were entered into the database using the International Classification of Diseases, Tenth Revision (ICD-10) codes. UCODs based on death certificates were defined as the diagnoses listed last in Part I of death certificates according to guidelines published by the Ministry of Health, Labour and Welfare in Japan.^16^ UCODs based on postmortem examination in conjunction with clinical information were diagnosed by the same two pathologists, M.S. and T.A., as the reference standard. UCODs specified for each subject were coded using Simcode as well as ICD-10. Simcode is the classification code developed by the Japanese Ministry of Health, Labour and Welfare to define vital statistics.^17^ The overall agreement between UCOD identified on death certificates and the reference standard was classified into the following categories: 1. Perfect ICD-10 code agreement; 2. Disagreement involving the same organ system; 3. Disagreement, but listed as a COD on death certificate; and 4. Complete disagreement. We defined these agreement proportions as the concordance rates, sensitivity as the proportion of the cases positively identified using both methods (UCOD identified on death certificate [+] and UCOD identified using the reference standard [+]) to the cases positively identified using the reference standard, and specificity as the proportion of the cases negatively identified using both methods (UCOD identified on death certificate [−] and UCOD identified using the reference standard [−]) to the cases negatively identified using the reference standard.

### Statistical analysis

McNemar’s test was used to evaluate differences between UCOD proportions estimated based on data solely from the death certificates and those estimated based on reference standard data. We also calculated the 95% Wald confidence intervals (CIs) with Bonett-Price Laplace adjustment for differences between proportions.^18^ Multivariate unconditional logistic regression analyses assessed the effect of age at death (<80 vs 80–89 and ≥90 years), sex, comorbidity, and major UCODs identified on death certificates (cancer, heart disease, pneumonia, and others) on UCOD misclassification. Comorbidity was defined as the number of clinical findings present among the 26 findings registered in the GEAD. In the logistic regression model, we had classified the number of comorbidity into three groups: no or low comorbidity (0–1 finding), moderate comorbidity (2–4 findings), and high comorbidity (≥5 findings).

Sensitivity and specificity with 95% Clopper-Pearson exact CIs were calculated for UCODs estimated to be present in at least 5% of the study population. We used SAS and JMP software for Windows (versions 9.3 and 10, respectively; SAS Institute, Cary, NC, USA) for all statistical analyses. Statistical significance was set at *P* < 0.05.

### Ethical considerations

The Japanese Postmortem Examination and Corpse Preservation Act generally permits use of autopsy materials for medical education and research. This study was approved by the ethics committee of Tokyo Metropolitan Geriatric Hospital (#240423).

## RESULTS

Table 1 shows subject characteristics. The average age at death was 79.8 years (range, 46–100 years; median, 80 years). Median number of major clinical findings was 3 (range, 0–8).

**Table 1.** Patient characteristics

Sex	Female (*n* = 187)	Male (*n* = 263)	Total (*n* = 450)
Mean (SD) age at death, years	81.9 (8.7)	78.2 (8.6)	79.8 (8.8)
frequency (%)			
<70 years	9 (5%)	33 (13%)	42 (9%)
70–79 years	61 (33%)	118 (45%)	179 (40%)
80–89 years	75 (40%)	83 (32%)	158 (35%)
≥90 years	42 (23%)	29 (11%)	71 (16%)

Mean (SD) number of major clinical findings	3.1 (1.7)	3.1 (1.6)	3.1 (1.7)
frequency (%)			
0–1	34 (18%)	50 (19%)	84 (19%)
2–4	117 (63%)	164 (62%)	281 (62%)
≥5	36 (19%)	49 (19%)	85 (19%)

UCOD distributions by sex are shown in Table 2. Simcodes generally conformed to ICD-10 codes, which are also shown in Table 2. The results indicate that cancer mortality would be underestimated (the absolute difference between death certificate information and the reference standard was 5.3% in women [95% CI, 0.49–10.0%; *P* = 0.025] and 6.1% in men [95% CI, 2.2–9.9%; *P* = 0.0017]), whereas the mortality for respiratory system diseases, especially pneumonia, would be overestimated (the absolute difference between death certificate information and the reference standard was 6.4% [95% CI, 1.6–11.1%; *P* = 0.0073] in women and 8.7% [95% CI, 4.1–13.3%; *P* = 0.0002] in men).

**Table 2.** Patients proportion of UCOD measured by death certificates only or by clinical and autopsy reports

Disease category	ICD-10 codes	Females (*n* = 187)			Males (*n* = 263)		
	
		UCOD on the death certificates	UCOD based on clinical and autopsy-derived information	absolute difference^a^	UCOD on the death certificates	UCOD based on clinical and autopsy-derived information	absolute difference^a^
**Certain infectious and parasitic diseases**	A00–B99	6 (3.2%)	4 (2.1%)	−1.1%	10 (3.8%)	11 (4.2%)	0.4%
**Malignant neoplasms**	C00–C97	**62 (33.2%)**	**72 (38.5%)**	**5.3%**	**94 (35.7%)**	**110 (41.8%)**	**6.1%**
Malignant neoplasms of lip, oral cavity, and pharynx	C00–C14	0 (0.0%)	0 (0.0%)	0.0%	0 (0.0%)	1 (0.4%)	0.4%
Malignant neoplasm of esophagus	C15	0 (0.0%)	0 (0.0%)	0.0%	1 (0.4%)	1 (0.4%)	0.0%
Malignant neoplasm of stomach	C16	1 (0.5%)	2 (1.1%)	0.6%	14 (5.3%)	17 (6.5%)	1.2%
Malignant neoplasm of colon	C18	4 (2.1%)	4 (2.1%)	0.0%	2 (0.8%)	2 (0.8%)	0.0%
Malignant neoplasm of rectum and rectosigmoid junction	C19–C20	1 (0.5%)	1 (0.5%)	0.0%	2 (0.8%)	2 (0.8%)	0.0%
Malignant neoplasm of liver and intrahepatic bile ducts	C22	2 (1.1%)	5 (2.7%)	1.6%	4 (1.5%)	5 (1.9%)	0.4%
Malignant neoplasm of gallbladder and unspecified parts of biliary tract	C23–C24	5 (2.7%)	8 (4.3%)	1.6%	3 (1.1%)	5 (1.9%)	0.8%
Malignant neoplasm of pancreas	C25	4 (2.1%)	3 (1.6%)	−0.5%	4 (1.5%)	5 (1.9%)	0.4%
Malignant neoplasm of trachea, bronchus, and lung	C33–C34	14 (7.5%)	13 (7.0%)	−0.5%	26 (9.9%)	31 (11.8%)	1.9%
Malignant neoplasm of cervix uteri, corpus uteri, and uterus	C53–C55	1 (0.5%)	1 (0.5%)	0.0%	—	—	—
Malignant neoplasm of prostate	C61	—	—	—	1 (0.4%)	0 (0.0%)	−0.4%
Malignant neoplasm of bladder	C67	3 (1.6%)	1 (0.5%)	−1.1%	1 (0.4%)	1 (0.4%)	0.0%
Malignant lymphoma	C81–C85	11 (5.9%)	13 (7.0%)	1.1%	8 (3.0%)	11 (4.2%)	1.2%
Leukemia	C91–C95	10 (5.3%)	16 (8.6%)	3.3%	23 (8.7%)	24 (9.1%)	0.40%
Other malignant neoplasms	Others in C00–C97	6 (3.2%)	5 (2.7%)	−0.5%	5 (1.9%)	5 (1.9%)	0.0%
**Non-malignant neoplasms**	D00–D48	**6 (3.2%)**	**1 (0.5%)**	**−2.7%**	**3 (1.1%)**	**5 (1.9%)**	**0.8%**
**Diseases of the blood and blood-forming organs and certain disorders involving the immune mechanism**	D50–D89	**1 (0.5%)**	**2 (1.1%)**	**0.6%**	**3 (1.1%)**	**3 (1.1%)**	**0.0%**
**Endocrine, nutritional, and metabolic diseases**	E00–E90	**1 (0.5%)**	**6 (3.2%)**	**2.7%**	**5 (1.9%)**	**5 (1.9%)**	**0.0%**
Diabetes mellitus	E10–E14	1 (0.5%)	2 (1.1%)	0.6%	2 (0.8%)	3 (1.1%)	0.3%
Other endocrine, nutritional, and metabolic diseases	Others in E00–E90	0 (0.0%)	4 (2.1%)	2.1%	3 (1.1%)	2 (0.8%)	−0.3%
**Mental and behavioral disorders**	F00–F99	**0 (0.0%)**	**1 (0.5%)**	**0.5%**	**0 (0.0%)**	**0 (0.0%)**	**0.0%**
**Diseases of the nervous system**	G00–G99	**5 (2.7%)**	**6 (3.2%)**	**0.5%**	**4 (1.5%)**	**7 (2.7%)**	**1.2%**
**Diseases of the circulatory system**	I00–I99	**46 (24.6%)**	**52 (27.8%)**	**3.2%**	**41 (15.6%)**	**45 (17.1%)**	**1.5%**
Hypertensive diseases	I10–I15	1 (0.5%)	0 (0.0%)	−0.5%	2 (0.8%)	0 (0.0%)	−0.8%
Heart disease	I01–I02, I05–I09, I20–I25, I27, I30–I52	26 (13.9%)	34 (18.2%)	4.3%	27 (10.3%)	33 (12.5%)	2.2%
Cerebrovascular diseases	I60–I69	9 (4.8%)	6 (3.2%)	−1.6%	4 (1.5%)	2 (0.8%)	−0.7%
Aortic aneurysm and dissection	I71	5 (2.7%)	6 (3.2%)	0.5%	4 (1.5%)	6 (2.3%)	0.8%
Diseases of the circulatory system other than aortic aneurysm and dissection	Others in I00–I99	5 (2.7%)	6 (3.2%)	0.5%	4 (1.5%)	4 (1.5%)	0.0%
**Diseases of the respiratory system**	J00–J99	**29 (16.5%)**	**17 (9.1%)**	**−7.4%**	**70 (26.6%)**	**51 (19.3%)**	**−7.3%**
Pneumonia	J12–J18	20 (10.7%)	8 (4.3%)	−6.4%	37 (14.1%)	14 (5.3%)	−8.8%
Chronic obstructive pulmonary disease	J41–J44	2 (1.1%)	0 (0.0%)	−1.1%	11 (4.2%)	11 (4.2%)	0.0%
Other diseases of the respiratory system	Others in J00–J99	7 (3.7%)	9 (4.8%)	1.1%	22 (8.4%)	26 (9.9%)	1.5%
**Diseases of the digestive system**	K00–K93	**16 (8.6%)**	**14 (7.5%)**	**−1.1%**	**15 (5.7%)**	**13 (4.9%)**	**−0.8%**
**Diseases of the skin and subcutaneous tissue**	L00–L99	**0 (0.0%)**	**1 (0.5%)**	**0.5%**	**1 (0.4%)**	**0 (0.0%)**	**−0.4%**
**Diseases of the musculoskeletal system and connective tissue**	M00–M99	**1 (0.5%)**	**5 (2.7%)**	**2.2%**	**1 (0.4%)**	**1 (0.4%)**	**0.0%**
**Diseases of the genitourinary system**	N00–N99	**7 (3.7%)**	**5 (2.7%)**	**−1.0%**	**4 (1.5%)**	**6 (2.3%)**	**0.8%**
**Other cause of death**	Others	**7 (3.7%)**	**1 (0.5%)**	**−3.2%**	**12 (4.6%)**	**6 (2.3%)**	**−2.3%**

UCOD, underlying cause of death.

^a^The difference between the proportion of UCOD based on clinical and autopsy-derived information and that of UCOD on the death certificates.

Of 450 UCODs identified on death certificates, 214 (47.6%) agreed completely with UCODs identified based on clinical and post-autopsy reports at ICD-10 three-digit code levels. When we applied Simcode (broader categories than the ICD-10 code categories shown in Table 2) to UCODs, the concordance rate increased to 59.3% and was further improved to 69.6% when major Simcodes (largest CODs category, indicated by boldface in Table 2, used for rough national mortality statistics) were used (Figure). Of 236 instances of UCOD disagreement, 83 (35.2%) cases were assigned to the same organ system, 38 (16.1%) were assigned as CODs but not UCODs on the death certificates, and 115 (48.7%) disagreed completely.

**Figure.**
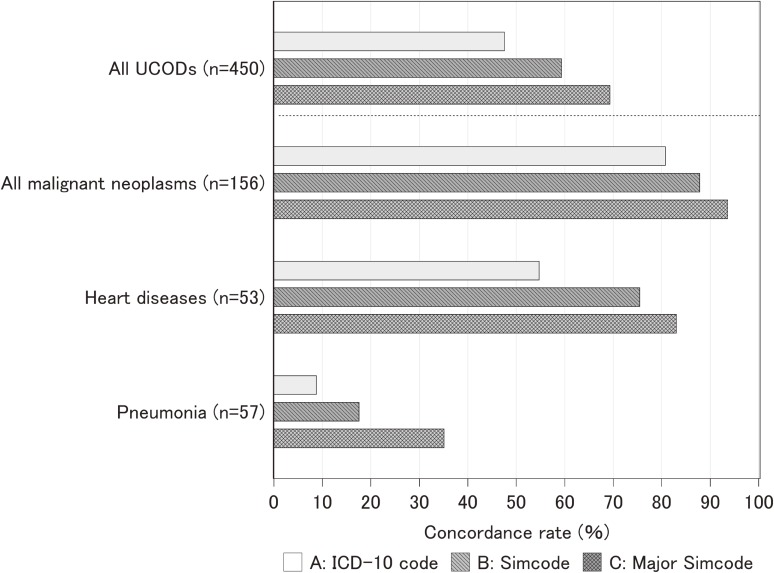
Concordance rates for UCOD recorded on the death certificates and judgment from clinical and pathological records by coding methods for CODs.

We also explored how concordance rates varied depending on UCODs. The concordance rate for cancer was 80.8% at the ICD-10 code level and increased to 93.6% at the major Simcode level. The concordance rate at the ICD-10 code level for heart disease was not high (54.7%); however, it improved to 83.0% at the major Simcode level. Among major UCODs, pneumonia, which is the third leading COD in Japan in 2012,^19^ had the lowest concordance rate (8.8% at the ICD-10 code level) (Figure).

We next examined the effects of sex, age, comorbidity, and UCODs on misclassification of UCODs identified on death certificates (Table 3). We found that sex, comorbidity, and age did not affect the UCOD misclassification rate (*P* = 0.53, *P* = 0.75, and *P* = 0.13, respectively), although the misclassification rate showed an increasing trend, especially for cases >90 years old (adjusted odds ratio [vs <80 years old] 1.44; 95% CI, 0.72–2.88). The strongest factor for misclassification was UCODs (*P* < 0.0001); the results also show that cancer and heart disease were less often misclassified than other minor UCODs (adjusted odds ratio 0.10; 95% CI, 0.06–0.16 and adjusted odds ratio 0.34; 95% CI, 0.18–0.65, respectively), whereas pneumonia was significantly misclassified compared to other minor UCODs (adjusted odds ratio 4.44; 95% CI, 1.66–11.8) (Table 3). On exploring the factors influencing accuracy of sensitivity and specificity for each disease, we found that age (>90 years) had a profound influence on specificity for pneumonia (odds ratio 3.23; 95% CI, 1.50–6.69; *P* = 0.0016), although the sample size was relatively small for such disease-specific analyses.

**Table 3.** Multivariate logistic regression analysis for agreement between UCODs evaluated by death certificates only and clinical and autopsy-based UCODs

Variables in the model	Adjusted OR	95% CI	*P* value^a^
Gender (female vs male)	1.16	0.73, 1.84	0.53

UCOD in death certificates			<0.0001
Cancer (vs others)	0.10	0.06, 0.16	<0.0001
Heart Disease (vs others)	0.34	0.18, 0.65	0.018
Pneumonia (vs others)	4.44	1.66, 11.8	<0.0001

Age			0.134
80–89 (vs <80) years	0.73	0.44, 1.21	0.050
≥90 (vs <80) years	1.44	0.72, 2.88	0.114

Number of clinical findings (Comorbidity)			0.75
2–4 (vs 0–1)	0.79	0.44, 1.45	0.59
≥5 (vs 0–1)	0.81	0.39, 1.70	0.76

CI, confidence interval; OR, odds ratio; UCOD, underlying cause of death.

^a^*P* value was from Wald Chi-Square test.

Finally, we evaluated the sensitivity and specificity of UCODs estimated to be present in at least 5% of the population (Table 4). Statistics were calculated for each UCOD identified on death certificates compared with the reference standard of assessment by two pathologists based on autopsy data and past clinical records. Overall, specificity for each UCOD was at least 85%. Sensitivity for any cancer was high (80%), although values varied according to organ. Sensitivity for heart disease was 60%, and sensitivity for pneumonia was very low (46%). Results also suggested that diseases of the digestive system were difficult to specify as UCOD (sensitivity, 51.9%). Among 13 deaths attributable to digestive diseases, 5 (38%) were reported as deaths due to unknown causes, 3 (23%) as deaths due to infectious diseases, and 3 (23%) as deaths due to heart disease.

**Table 4.** Sensitivity and specificity of major UCODs evaluated by death certificates only

UCOD	*n* of UCOD on the death certificates	*n* of UCOD based on clinical and autopsy-derived information	*n* of both UCODs truly classified (+)	Sensitivity (%)		Specificity (%)	
	
				Point estimate	95% CI	Point estimate	95% CI
**Certain infectious and parasitic diseases**	16	15	6	40.0	16.3, 67.7	97.7	95.8, 98.9
**Malignant neoplasms**	**156**	**182**	**146**	80.2	73.7, 85.7	96.3	93.3, 98.2
Stomach	15	19	14	73.7	48.8, 90.9	99.8	98.7, 100
Trachea, bronchus, and lung	40	44	38	86.4	72.7, 94.8	99.5	98.2, 99.9
Malignant lymphoma	19	24	18	75.0	53.3, 90.2	99.8	98.7, 100
Leukemia	33	40	30	75.0	58.8, 87.3	99.3	97.9, 99.9
**Diseases of the circulatory system**	**87**	**97**	**74**	71.1	61.1, 79.9	94.9	92.1, 97.0
Heart disease	53	67	40	59.7	47.0, 71.5	96.6	94.3, 98.2
**Diseases of the respiratory system**	**99**	**68**	**48**	70.6	58.3, 81.0	86.7	82.8, 89.9
Pneumonia	57	22	10	45.5	24.4, 67.8	89.0	85.7, 91.8
**Diseases of the digestive system**	**31**	**27**	**14**	51.9	32.0, 71.3	96.0	93.6, 97.6

CI, confidence interval; UCOD, underlying cause of death.

Table 5 also shows that deaths due to cancer and heart disease were underestimated regardless of true UCODs, and 18 (38%) of 47 deaths due to pneumonia and 28 (55%) of 51 deaths due to respiratory diseases would be considered deaths due to cancer or heart disease.

**Table 5.** List of major UCODs misclassified on death certificates

UCOD specified with the death certificate	UCOD specified with clinical and autopsy records									
**Certain infectious and parasitic diseases (*n* = 10)**	Diseases of the digestive system (3)	Diseases of the respiratory system other than pneumonia (2)	Cancer (2)	Pneumonia (1)	Diseases of the genitourinary system (1)	Diseases of the skin and subcutaneous tissue (1)				
**Malignant neoplasms (*n* = 10)**	Heart disease (3)	Diseases of the genitourinary system (2)	Non-malignant neoplasms (1)	Diseases of the blood and blood-forming organs and certain disorders involving the immune mechanism (1)	Diseases of the circulatory system other than aortic aneurysm and dissection (1)	Pneumonia (1)	Diseases of the respiratory system other than pneumonia (1)			
Stomach (*n* = 1)	Pneumonia (1)									
Trachea, bronchus, and lung (*n* = 2)	Diseases of the blood and blood-forming organs and certain disorders involving the immune mechanism (1)	Heart disease (1)								
Malignant lymphoma (*n* = 1)	other cancer (1)									
Leukemia (*n* = 3)	other cancer (1)	Non-malignant neoplasms (1)	Diseases of the genitourinary system (1)							
**Diseases of the circulatory system (*n* = 13)**	Diseases of the digestive system (4)	Cancer (3)	Certain infectious and parasitic diseases (2)	Pneumonia (2)	Diseases of the respiratory system other than pneumonia (2)	unknown (2)	Diseases of the blood and blood-forming organs and certain disorders involving the immune mechanism (1)	Diabetes mellitus (1)	Diseases of the genitourinary system (1)	
Heart disease (*n* = 13)	Diseases of the circulatory system other than heart disease (4)	Diseases of the digestive system (3)	Certain infectious and parasitic diseases (1)	Diseases of the blood and blood-forming organs and certain disorders involving the immune mechanism (1)	Diabetes mellitus (1)	Pneumonia (1)	Diseases of the genitourinary system (1)	unknown (1)		
**Diseases of the respiratory system (*n* = 51)**	Cancer (17)	Heart disease (11)	Certain infectious and parasitic diseases (6)	Endocrine, nutritional and metabolic diseases other than diabetes (4)	Diseases of the nervous system (4)	Diseases of the musculoskeletal system and connective tissue (3)	Non-malignant neoplasms (2)	unknown (2)	Cerebrovascular diseases (1)	Diseases of the genitourinary system (1)
Pneumonia (*n* = 47)	Diseases of the respiratory system other than pneumonia (10)	Cancer (9)	Heart disease (9)	Certain infectious and parasitic diseases (4)	Endocrine, nutritional and metabolic diseases other than diabetes (4)	Diseases of the nervous system (4)	Diseases of the musculoskeletal system and connective tissue (2)	Cerebrovascular diseases (1)	Diseases of the genitourinary system (1)	unknown (1)
**Diseases of the digestive system (*n* = 17)**	cancer (5)	Heart disease (4)	Non-malignant neoplasms (2)	Pneumonia (2)	Diseases of the genitourinary system (2)	Diseases of the respiratory system other than pneumonia (1)	unknown (1)			

UCOD, underlying cause of death.

## DISCUSSION

We evaluated the accuracy of UCODs, particularly major UCODs, recorded on the death certificates of elderly patients in Japan. To our knowledge, this is the first report to quantitatively estimate accuracy for several UCODs specified on death certificates. Data from death certificates are used for many clinical and population-based studies and national vital statistics, although the difficulties in properly completing the COD section of the death certificate to ensure accuracy of COD data have been well documented.^1^^–^^5^ Several recently proposed statistical methods to account for outcome variable misclassification enable bias correction of effect estimates due to misclassified outcomes, such as those measured by death certificates.^20^^–^^23^ However, it is difficult to quantitatively evaluate the accuracy of data from death certificates, as we have done here, because reference standard data is not easily obtainable, especially in studies that utilize large national databases. Our results might be informative either for applying bias correction methods or sensitivity analyses to assess effect estimate bias in studies using data from death certificates.

According to national vital statistics’ reports, the four leading UCODs in Japan in 2000 were malignant lymphoma (29.6% of deaths among 80- to 84-year-olds), heart disease (16.1% of deaths among 80- to 84-year-olds), cerebrovascular disease (11.6% of deaths among 80- to 84-year-olds), and pneumonia (11.4% of deaths among 80- to 84-year-olds). In our study, the top four UCODs were malignant lymphoma (28.5% among individuals in their 80s), pneumonia (16.5% among individuals in their 80s), heart disease (13.3% among individuals in their 80s), and digestive system disease (7.6% among individuals in their 80s). Thus, except for death due to cerebrovascular disease, the distribution of UCODs in our population was similar. This is because the Tokyo Metropolitan Geriatric Hospital is not an acute care hospital, and most cases had chronic diseases. The population analyzed here is not representative of the whole population of elderly people in Japan, and we could not assess the accuracy of UCODs for acute diseases in this study. However, our data showed that deaths due to cancer and heart disease based solely on death certificate records would be underestimated, a finding that has also been reported in previous studies.^9^^,^^12^ Hu et al assessed the reliability of COD for the Surveillance, Epidemiology, and End Results (SEER) database using a relative survival approach and showed that the number of cancer-specific deaths documented in SEER was over-coded for early stage cancers or cancers with favorable prognoses, whereas SEER tended to undercode the number of cancer-specific deaths for cancers with generally poor prognosis or advanced-stage cancers.^24^ In our study data, most cancer-specific deaths were of poor prognosis or advanced-stage cancer, so our observation is consistent with previous research.

In general, COD in elderly patients is subject to speculation because of the competing effects of comorbidity-associated mortality. However, while our data showed neither significant comorbidity nor age effects, we observed that the misclassification rate in very old patients tended to be higher than in younger patients even after adjusting for UCOD and comorbidity. This suggests that “more likely” CODs without detailed investigation are recorded on death certificates regardless of patient history, particularly if the patient was more than 90 years of age and died of old age.

To our knowledge, there have been no previous reports on the accuracy of COD data from death certificates for pneumonia, despite being a leading COD in many countries. As discussed above, the UCOD recorded for elderly patients could be the “more likely” COD, and pneumonia would be a most likely UCOD in very elderly patients because many of them are likely to die of pneumonia. Another reason for the high pneumonia misclassification rate was that many cases of aspiration pneumonia were reported as deaths due to pneumonia. In contrast to the misclassified cases of death due to digestive or other minor diseases, misclassified death due to pneumonia is likely to be caused by misjudgment and not by errors in diagnostic techniques. Myers et al showed that the accuracy of death certificates could be improved by implementation of a simple educational intervention.^25^ In Japan, many medical doctors previously reported heart failure as the UCOD on death certificates regardless of the true UCOD.^12^^,^^26^ However, this poor practice has improved in the past several decades by adding a note on death certificates according to a revised ICD-10 code, which states, “Do not enter the mode of dying, such as cardiac or respiratory arrest, shock, or heart failure.” Therefore, the pneumonia misclassification rate could be reduced by education or by including notes or instructions in the guidelines for completing death certificates when pneumonia appears as a condition on the death certificate.

### Study limitations

Although having multiple-cause autopsy mortality data was a strength of this study, the potential for autopsy bias limits our ability to generalize the results to the rest of the population. As mentioned above, we could not assess the accuracy of UCODs for acute diseases, such as cerebrovascular death. Additionally, we were unable to measure the accuracy of UCOD for minor diseases and diseases for which only clinical diagnoses were available, such as diabetes or some psychiatric diseases. To assess the validity of death certificate data for such diseases, additional disease-specific studies modeled on previous reports are necessary.^3^^,^^4^^,^^27^ The data we investigated were collected more than 10 years ago. If the medical record training for doctors had been well-established during the period, we might have obtained more accurate sensitivities and specificities. However, to our knowledge, the situation has not changed much, so improvements in medical recordkeeping may have little effect on the interpretation of our results.

### Conclusion

Researchers should be aware of the accuracy of COD data on death certificates used as research resources, particularly for elderly research subjects who died from diseases other than cancer (especially pneumonia).

## ONLINE ONLY MATERIAL

Abstract in Japanese.
