# Supplementary material for: Accuracy of Death Certificates and Assessment of Factors for Misclassification of Underlying Cause of Death
Source: J Epidemiol. 2016 Apr 5;26(4):191–8. doi: 10.2188/jea.JE20150010 (PMC4808686; doi:10.2188/jea.JE20150010)
Supplement: Abstract in Japanese. [file je-26-191-s001.pdf]

# 死亡診断書の正確度と原死因の誤分類に関連する要因に関する検討

三重野牧子[1], 田中紀子[2], 新井冨生[3], 川原拓也[4], 口羽文[5], 石川鎮清[6], 沢辺元司[7]
[1]自治医科大学 情報センター・医学情報学, [2]国立国際医療研究センター臨床研究センター医療情報解析研究部医学統計研究室, [3]東京都健康長寿医療センター病理診断科, [4]東京大学大学院医学系研究科公共健康科学専攻生物統計学教室, [5]国立がん研究センター研究支援センター生物統計部, [6]自治医科大学 医学教育センター
7. 東京医科歯科大学保健衛生学研究科生体検査科学専攻分子病態検査学講座

**背景：**死亡票から得られた死因はしばしば不正確かつ不完全である。しかしながら、死亡票に記録された原死因の正確性について、特に多疾患を含む場合には網羅的に検討されてきていない。
**方法：**日本国内のある老年病専門病院で 2000 年 2 月から 2002 年 8 月に行われた連続剖検 450 例を対象とした。死亡票で報告された主な原死因（悪性腫瘍、心疾患、肺炎）を、剖検データと臨床カルテ記録に基づいて病理学者が判断した参照基準と比較し、一致率、感度、特異度について評価した。性、年齢、併存疾患、原死因の誤分類への影響を評価するため、ロジスティック回帰分析を行った。
**結果：**悪性腫瘍についての一致率は 81%と比較的高い結果であったが、心疾患では 55%、肺炎では 9%と低い一致率を示した。全体の一致率は 48%であった。性と併存疾患は原死因の誤分類率に影響しなかった。また、統計的有意ではなかったものの、患者年齢が上がるにつれて誤分類率は上昇する傾向がみられた。誤分類に対するもっとも強い要因は原死因であった($p<0.0001$)。悪性腫瘍についての感度は 80%、特異度は 96%と高かった一方で、心疾患と肺炎の感度はそれぞれ 60%と 46%であった。各原死因についての特異度は 85%以上を示していた。
**結論：**研究者は、高齢の肺炎患者を対象とした場合は特に、研究資源として用いた死亡票の死因データの正確性に注意を払うべきである。
